# Tubule-Derived Follistatin Is Increased in the Urine of Rats with Renal Ischemia and Reflects the Severity of Acute Tubular Damage

**DOI:** 10.3390/cells12050801

**Published:** 2023-03-04

**Authors:** Izumi Nagayama, Kaori Takayanagi, Hajime Hasegawa, Akito Maeshima

**Affiliations:** Department of Nephrology and Hypertension, Saitama Medical Center, Saitama Medical University, Kawagoe 350-8550, Japan

**Keywords:** follistatin, acute kidney injury, ischemia reperfusion

## Abstract

Activin A, a member of the TGF-beta superfamily, is a negative regulator of tubular regeneration after renal ischemia. Activin action is controlled by an endogenous antagonist, follistatin. However, the role of follistatin in the kidney is not fully understood. In the present study, we examined the expression and localization of follistatin in normal and ischemic rat kidneys and measured urinary follistatin in rats with renal ischemia to assess whether urinary follistatin could serve as a biomarker for acute kidney injury. Using vascular clamps, renal ischemia was induced for 45 min in 8-week-old male Wistar rats. In normal kidneys, follistatin was localized in distal tubules of the cortex. In contrast, in ischemic kidneys, follistatin was localized in distal tubules of both the cortex and outer medulla. Follistatin mRNA was mainly present in the descending limb of Henle of the outer medulla in normal kidneys but was upregulated in the descending limb of Henle of both the outer and inner medulla after renal ischemia. Urinary follistatin, which was undetectable in normal rats, was significantly increased in ischemic rats and peaked 24 h after reperfusion. There was no correlation between urinary follistatin and serum follistatin. Urinary follistatin levels were increased according to ischemic duration and were significantly correlated with the follistatin-positive area as well as the acute tubular damage area. These results suggest that follistatin normally produced by renal tubules increases and becomes detectable in urine after renal ischemia. Urinary follistatin might be useful to assess the severity of acute tubular damage.

## 1. Introduction

Activins are pleiotropic factors belonging to the transforming growth factor-beta (TGF-beta) superfamily and regulate cell growth and differentiation in many organs [1,2]. The expression of activin A is undetectable in normal kidneys but is upregulated in tubular cells of the kidney after renal ischemia in both mice [3] and rats [4]. Activin A inhibits tubular regeneration by modulating the expression of Pax-2, a critical factor for kidney development, after renal ischemia [5]. Activin A significantly inhibits cell growth and induces apoptosis in cultured proximal tubular cells (LLC-PK1) [6]. Furthermore, activin A produced by tubular cells activates renal interstitial fibroblasts in a paracrine manner during the fibrotic processes of the kidney [7,8]. These data suggest that activin A functions as a negative regulator of tubular regeneration after kidney injury as well as a potent activator of renal fibrosis [9,10].

In various tissues, local activin A effects are modulated by its endogenous antagonist follistatin [11]. Follistatin was first isolated from ovarian follicular fluid as a specific inhibitor of follicle stimulating hormone [12,13]. Through neutralization of activin A, follistatin participates in various processes such as cell growth, development, and differentiation [14]. For example, previous studies demonstrated that follistatin administration attenuates ischemic diseases including myocardial and hepatic ischemia-reperfusion injury (IRI) [15,16]. In the kidney, exogenous administration of recombinant follistatin has been reported to reduce renal damage by blockade of endogenous activin A effects in models of renal ischemia/reperfusion [4], unilateral ureteral obstruction [8], and cisplatin nephropathy [17]. A previous report showed that follistatin is ubiquitously expressed in rat tissues including the kidney [18]. However, the precise distribution of follistatin in normal and damaged kidneys is not fully known. To address this issue, we examined the localization of follistatin mRNA and protein in normal and ischemic rat kidneys and assessed whether follistatin is detectable in urine of rats with renal ischemia in the present study.

## 2. Materials and Methods

### 2.1. Experimental Protocols

Wistar rats (male, 8-week-old) were obtained from Japan Charles River (Yokohama, Japan). Rats under specific pathogen-free “SPF” conditions were provided with autoclaved food and sterile water ad libitum. Renal ischemic injury was induced in rats as described previously [4]. Briefly, under general anesthesia with 1.5% isoflurane, renal ischemia was induced by clamping both renal arteries for 45 min using a non-traumatic vascular clamp. Core body temperature was maintained at 37 °C by placing the rats on a homoeothermic table. After the ischemia periods, the clamps were removed, and reperfusion of the kidneys was confirmed visually. The rats were subsequently sacrificed after various post-ischemic periods (*n* = 6 in each group). The kidneys were removed and frozen for RNA extraction or fixed in 10% formalin for routine paraffin embedding and sectioning for histologic analysis. Urine was collected from individual rats housed in metabolic cages. After centrifugation at 10,000 rpm for 5 min, all urine samples were stored at −80 °C until the day of analysis. Sham operations (*n* = 8) were performed in a similar manner, except for clamping of the renal arteries. Blood samples were obtained at the time of death, and serum samples were maintained at −20 °C until measurements. All animal procedures were approved by and conducted in accordance with the Fundamental Guidelines of the Ethics Review Committee for Animal Experimentation of Saitama Medical University (approval number 3117).

### 2.2. Measurement of Renal Function

Urinary or serum creatinine and blood urea nitrogen (BUN) levels were measured by a standard method at Oriental Kobo Life Science Laboratory (Nagahama, Japan).

### 2.3. Immunohistochemical Analysis

Immunostaining was performed using the DAKO EnVision labelled polymer system (Agilent Technologies, Inc, Santa Clara, CA, USA). Briefly, paraffin-embedded sections (3 μm-thick) were deparaffinized, hydrated according to standard methods; soaked in protein block serum-free solution (Dako); and incubated with primary antibody overnight at 4 °C. After washing with Tris-buffered saline containing 0.1% Tween 20 (TBS-T), sections were incubated with a peroxidase-conjugated secondary antibody followed by diaminobenzidine and then counterstained with hematoxylin. Following staining, the slides were photographed under a light microscope BX-61 (Olympus Optical Co., Ltd., Tokyo, Japan) and analyzed. For the immunostaining control, the primary antibody was replaced with PBS, which did not show positive signals, confirming specificity. Quantification of follistatin-positive areas was performed by measurement of positive areas in five randomly selected fields of the kidney at ×100 magnification using ImageJ software (National Institutes of Health, Bethesda, MD, USA).

The primary antibodies used in this study are as follows: mouse anti-follistatin antibody (60060-1-ig; Proteintech, Rosemont, IL, USA), rabbit anti-inhibin βA antibody (ab97705; Abcam, Cambridge, UK), rabbit anti-Na-Cl co-transporter (NCC) antibody (ab3553; Abcam), mouse anti-aquaporin (AQP)-1 antibody (sc-25287), mouse anti- aquaporin (AQP)-2 antibody (sc-515770), mouse anti-megalin (G-9) antibody (sc-515750; Santa Cruz Biotechnology, Santa Cruz, CA, USA), rabbit anti-uromodulin antibody (HPA043420; Sigma-Aldrich, St. Louis, MO, USA), goat anti-kidney injury molecule (KIM)-1 antibody (AF3689), and goat anti-neutrophil gelatinase-associated lipocalin (NGAL) antibody (AF1857; R&D Systems, Minneapolis, MN, USA).

### 2.4. Histological Examination

Semi-quantitative analysis of the tubule damage area was performed as described previously [4].

### 2.5. ELISA

Urinary and serum rat follistatin (LS-F21531; LSBio, Seattle, WA, USA), urinary rat KIM-1 (DY3689), urinary rat NGAL (DY3508), and urinary rat L-FABP (DY1565; R&D Systems) were measured by ELISA according to the manufacturer’s instructions.

### 2.6. In Situ Hybridization

In situ hybridization (ISH) was performed using an ISH Reagent Kit from Genostaff Co., Ltd. (Tokyo, Japan) and a DIG Nucleic Acid Detection Kit from Roche (Basel, Switzerland).

Probes for in situ hybridization were obtained from Genostaff Co., Ltd. (Tokyo, Japan). Sections were deparaffinized, rehydrated, and fixed in 4% paraformaldehyde (PFA) in PBS at room temperature (RT) for 10 min. After digestion with proteinase K (5 μg/mL) at 37 °C for 10 min, sections were postfixed in 4% PFA in PBS at RT for 15 min. Hybridization was performed with sense or antisense probes (0.5 ng/μL) at 58 °C for 16 h. Slides were then rinsed once in G-Wash at 60 °C for 10 min, 50% formamide in G-Wash at 60 °C for 10 min, washed twice in G-Wash at 60 °C for 20 min, and washed twice at 0.1x G-Wash at 60 °C for 20 min. Slides were incubated in 1× blocking solution for 60 min at RT and then incubated in a 1:5000 diluted solution of AP-conjugated anti-digoxigenin antibody for 60 min. After washing, the signals were detected with nitroblue tetrazolium chloride and 5-bromo-4-chloro-3-indolyphosphate.

### 2.7. Real-Time PCR

Kidneys were homogenized using a microhomogenizer and total RNA was extracted with ISOGEN (Nippon Gene, Toyama, Japan). First-strand cDNA was prepared using SuperScript III First-strand (Invitrogen, Carlsbad, CA, USA) according to the manufacturer’s instructions. Real-time PCR was performed using the StepOne Plus Real-time PCR System (Applied Biosystems, Foster City, CA, USA). Reactions included 5 μL of a SYBR Green Real-time PCR Master Mix (TOYOBO, Osaka, Japan), 0.5 μL of 3′ primer, 0.5 μL of 5′ primer, and 1 μL of cDNA. Samples were incubated at 50 °C for 2 min, then at 95 °C for 2 min, followed by 49 cycles of 15 s at 95 °C, 15 s at 58 °C, and 60 s at 72 °C. The expression of each gene was quantified in separate tubes with the following primers: rat follistatin (154 bp) sense 5′-AAAACCTACCGCAACGAATG-3′, antisense 5′-AGGCATTATTGGTCTGATCC-3′; and rat GAPDH (130 bp) sense, 5′-CTACCCACGGCAAGTTCAAT-3%’, antisense 5′-TACTCAGCACCAGCATCACC-3′. Values were presented as relative expression normalized to GAPDH.

### 2.8. Statistical Analysis

Statistical analyses were performed using GraphPad Prism 8.3 (GraphPad software, San Diego, CA, USA). The Shapiro–Wilk test was used for testing normality. For two group comparisons, Student’s t-test was used for normally distributed data and the Mann–Whitney test or Wilcoxon test was used for skewed data. The Kruskal–Wallis and Dunn’s multiple comparison tests were used to compare the means of more than two variables. Spearman‘s rank correlation coefficient was used to analyze the correlations. Values of *p* < 0.05 were considered significant.

## 3. Results

### 3.1. Localization of Follistatin in Normal and Ischemic Rat Kidneys

**Figure 1:**
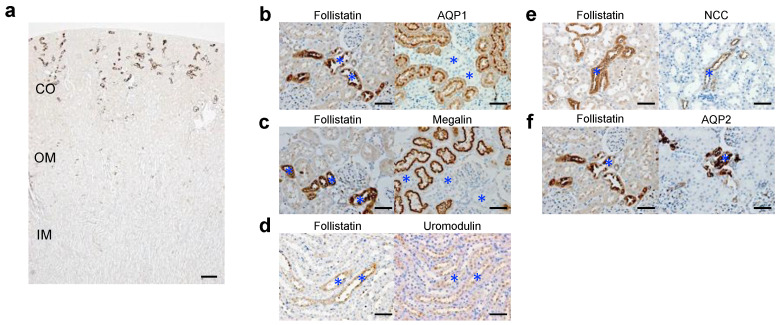
Localization of the follistatin protein in normal rat kidneys. (**a**) Localization of follistatin (brown) in normal rat kidneys was examined by immunostaining. Cortex, CO; outer medulla, OM; inner medulla, IM. Bar = 0.2 mm. (**b**–**f**) Identification of follistatin-positive tubules in rat normal kidneys using serial sections. Aquaporin 1, AQP1; Na-Cl co-transporter, NCC; aquaporin 2, AQP2. Asterisks indicate identical tubules. Bar = 50 μm.

We first examined the localization of follistatin in normal kidneys by immunostaining. Follistatin was present in renal tubules of the cortex (Figure 1a). Immunostaining using serial sections showed that follistatin-positive renal tubules were negative for AQP1 (Figure 1b) and megalin (Figure 1c) but positive for uromodulin (Figure 1d), NCC (Figure 1e), and AQP2 (Figure 1f). These data suggest that follistatin was localized in distal tubules and collecting ducts of cortex in normal rat kidneys.

**Figure 2:**
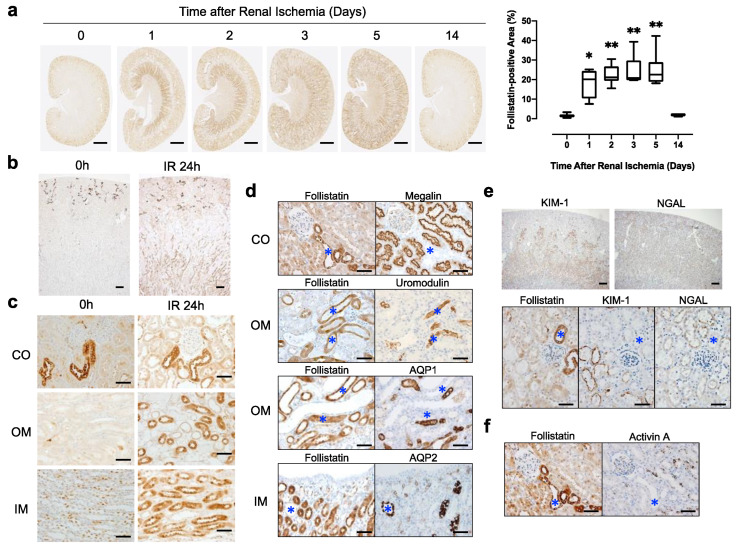
Localization of the follistatin protein in kidneys after renal ischemia. (**a**) Localization of follistatin (brown) in kidneys after renal ischemia was examined by immunostaining (left panel). Bar = 2 mm. Quantitative analysis of follistatin-positive area (right panel). Five randomly selected fields of the kidney were assessed at ×100 magnification. The follistatin-positive area was measured by ImageJ. Values are the means ± S.E. (*n* = 6–8). * *p* < 0.05, ** *p* < 0.01 vs. 0 h (**b**,**c**) Localization of the follistatin protein in normal and ischemic kidneys. The kidneys were collected at 0 h and 24 h after reperfusion. Ischemia reperfusion, IR. Bar = 0.2 mm in (**b**), 50 μm in (**c**). Cortex, CO; outer medulla, OM; inner medulla, IM. Bar = 50 μm. (**d**) Localization of follistatin protein and megalin, aquaporin 1 (AQP1), uromodulin (UMOD), or aquaporin 2 (AQP2) in ischemic kidneys was evaluated using serial sections. Cortex, CO; outer medulla, OM; inner medulla, IM. Bar = 50 μm. (**e**) Localization of follistatin, neutrophil gelatinase-associated lipocalin (NGAL), and kidney injury molecule-1 (KIM-1) in ischemic kidneys was evaluated using serial sections. Bar = 0.2 mm (upper panels), 50 μm (lower panels). (**f**) Localization of follistatin and activin A in ischemic kidneys was evaluated using serial sections. Bar = 50 μm. Asterisks indicate identical tubules.

We next examined the localization of follistatin in ischemic kidneys. Acute kidney injury was induced in rats by renal ischemia for 45 min. Low magnification images revealed that the follistatin-positive area increased transiently in the kidney after renal ischemia (Figure 2a). Quantitative analysis demonstrated that the follistatin-positive area was significantly increased in the kidney after renal ischemia (Figure 2a). Follistatin, which was detected in the cortex of normal kidneys, was increased in ischemic kidneys at 24 h after reperfusion (Figure 2b). Follistatin was detected in renal tubules not only in the cortex but also in the outer and inner medulla of ischemic kidneys (Figure 2c). Immunostaining using serial sections revealed that follistatin-positive tubules in the cortex were negative for megalin (Figure 2d). In contrast, follistatin-positive thin tubular cells in the outer medulla were positive for uromodulin and negative for AQP1 (Figure 2d). Thin tubular cells expressing follistatin in the inner medulla were negative for AQP2 in ischemic kidneys (Figure 2d). These data suggest that follistatin was increased in distal tubules of the outer medulla as well as in the loop of Henle of the inner medulla after renal ischemia.

We then compared the localization of follistatin and acute kidney injury (AKI) biomarkers such as KIM-1 and NGAL in ischemic kidneys. Expression of both KIM-1 and NGAL was detected in tubular cells of ischemic kidneys (Figure 2e). Immunostaining using serial sections revealed that follistatin was not co-localized with KIM-1 or NGAL in ischemic kidneys at 24 h after reperfusion (Figure 2e). Follistatin is a local modulator of activin A effects in various organs. We also compared the localization of follistatin and activin A in ischemic kidneys and found that follistatin and activin A were not co-localized (Figure 2f).

### 3.2. Expression and Localization of Follistatin mRNA in Normal and Ischemic Rat Kidneys

**Figure 3:**
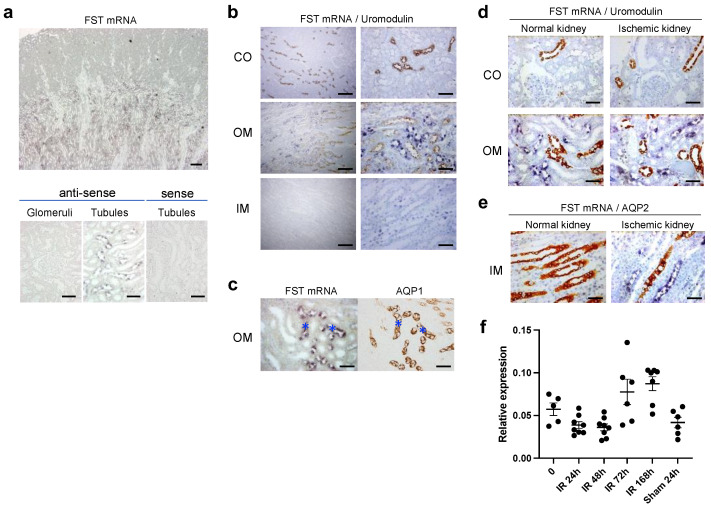
Expression and localization of follistatin mRNA in normal and ischemic rat kidneys. (**a**) Localization of follistatin mRNA in normal kidneys was examined by in situ hybridization. Hybridization signals are shown as blue color. Bar = 0.2 mm (left panel), 50 μm (right panels). (**b**) Follistatin mRNA (blue) and uromodulin (brown) in normal kidneys was evaluated by double staining. Cortex, CO; outer medulla, OM; inner medulla, IM. Bar = 0.2 mm (left panel), 50 μm (right panels). (**c**) Localization of follistatin mRNA and aquaporin 1 (AQP1) in normal rat kidneys was evaluated using serial sections. Asterisks indicate identical tubules. (**d**) Follistatin mRNA (blue) and uromodulin (brown) in normal and ischemic kidneys at 24 after reperfusion was evaluated by double staining. Cortex, CO; outer medulla, OM. (**e**) Follistatin mRNA (blue) and aquaporin 2 (AQP2, brown) in normal and ischemic kidneys was evaluated by double staining. Inner medulla, IM. Bar = 50 μm in (**c**–**e**). (**f**) Quantitative analysis of follistatin mRNA expression by real-time PCR. Values (relative expression ratio to GAPDH) are means  ±  S.E. (*n*  =  5–8).

Next, we assessed the distribution of follistatin mRNA in normal kidneys by in situ hybridization. Follistatin mRNA was observed in the outer medulla of normal rat kidneys (Figure 3a). Hybridization signals were detected in nuclei of renal tubules, but not in the glomeruli. A control experiment with a sense probe revealed no hybridization signal (Figure 3a). A combination of in situ hybridization and immunostaining showed that follistatin mRNA was not expressed in renal tubules positive for uromodulin (Figure 3b) but was co-localized in thin tubular cells positive for AQP1 in the outer medulla (Figure 3c), suggesting that follistatin mRNA was expressed in the descending loop of Henle of the outer medulla in normal rat kidneys.

We next assessed the localization of follistatin mRNA in ischemic kidneys at 24 h after reperfusion. Follistatin mRNA was observed in tubular cells negative for uromodulin in the outer medulla of both normal and ischemic kidneys (Figure 3d). Follistatin mRNA was distributed in thin tubular cells of ischemic kidneys, which were negative for AQP2 (Figure 3e), suggesting that the expression of follistatin mRNA was upregulated in the descending loop of Henle in the inner medulla after renal ischemia.

We also measured the mRNA expression level of follistatin in the kidneys after renal ischemia by real-time PCR. There were no significant differences in the expression level of follistatin between normal and ischemic kidneys (Figure 3f).

### 3.3. Urinary Follistatin Level in Rats with Renal Ischemia

**Figure 4:**
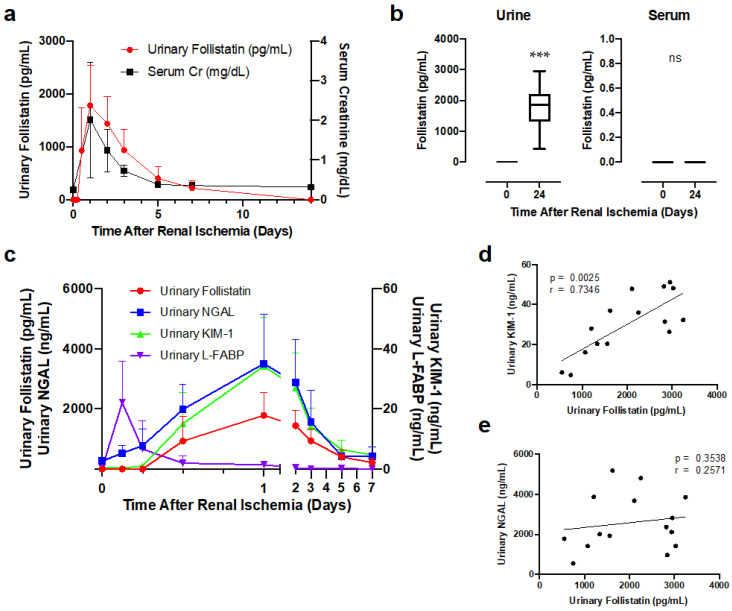
Urinary follistatin levels significantly increased after renal ischemia. (**a**) Time course changes in urinary follistatin and serum creatinine levels after reperfusion (*n* = 8). (**b**) Urinary and serum follistatin levels in normal and renal ischemia (45 min) rats were measured by ELISA. Values are means ± S.E. (*n* = 8). *** *p* < 0.001; N.S., not significant. (**c**) Time course changes in urinary follistatin, urinary NGAL, KIM-1, and L-FABP after reperfusion. (**d**,**e**) Correlations between urinary follistatin and urinary NGAL (**d**) or urinary KIM-1 (**e**) at 24 h after reperfusion. Renal ischemia (45 min) was performed, and urine was collected 24 h after reperfusion.

We further tested if urinary follistatin became detectable in rats with AKI. Renal ischemia was induced in rats and urinary follistatin levels were measured at the indicated time-points after reperfusion by ELISA. No follistatin was found in the urine of normal rats. In contrast, urinary follistatin became detectable at 12 h, peaked at 24 h after reperfusion, and decreased thereafter, which reflected serum creatinine level (Figure 4a).

The molecular weight of follistatin ranges from 31 to 39 kDa, raising the possibility that circulating follistatin is increased, filtered by the glomerulus, and then excreted into the urine after renal ischemia. To test this possibility, we measured serum follistatin levels 24 h after reperfusion, the time-point at which urinary follistatin peaked after renal ischemia. Urinary follistatin 24 h after renal ischemia was significantly higher than that at 0 h after renal ischemia (Figure 4b). Serum follistatin was undetectable in both normal and ischemic rats (Figure 4b), suggesting that urinary follistatin originates from the kidney rather than the blood.

We then compared the time course of changes in urinary follistatin with other AKI biomarkers. Urinary NGAL was elevated at 3 h, peaked at 24 h after reperfusion, and decreased thereafter (Figure 4c). Urinary L-FABP was elevated at 3 h and rapidly decreased thereafter. Urinary follistatin was detectable at 12 h and peaked at 24 h after reperfusion, which reflected the results for urinary KIM-1 (Figure 4c). We also analyzed the correlation of urinary follistatin levels with urinary NGAL and urinary KIM-1 at 24 h after reperfusion. Urinary follistatin was significantly correlated with urinary KIM-1 (Figure 4d) but not urinary NGAL (Figure 4e).

### 3.4. Correlation of Urinary Follistatin with Severity of Acute Tubular Damage in Ischemic Rats

**Figure 5:**
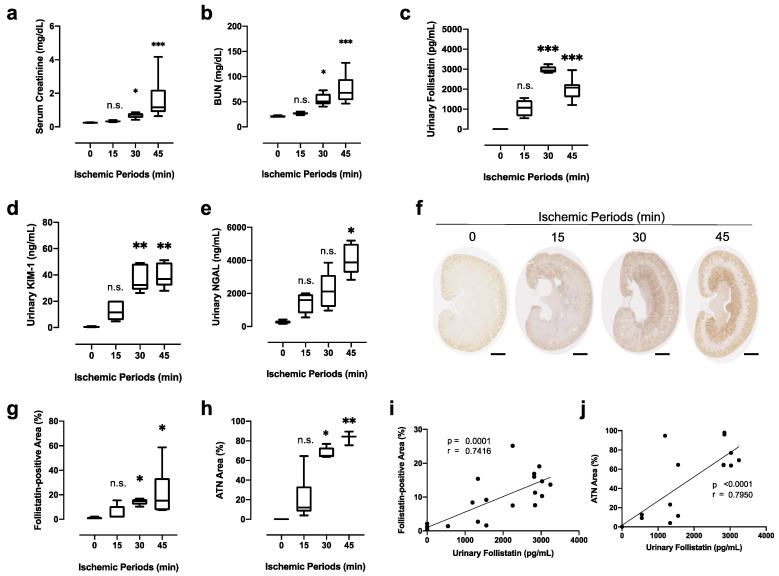
Correlation between urinary follistatin and severity of kidney damage. (**a**,**b**) Serum creatinine and BUN in rats with renal ischemia at 24 h after reperfusion. N.S., not significant, * *p* < 0.05, *** *p* < 0.001 vs. 0 h. (**c**–**e**) Urinary follistatin, urinary NGAL, and urinary KIM-1 in rats with renal ischemia for the indicated periods at 24 h after reperfusion. N.S., not significant, * *p* < 0.05, ** *p* < 0.01, *** *p* < 0.001 vs. 0 h. (**f**) Localization of follistatin (brown) in kidneys after renal ischemia for 15, 30, and 45 min was examined by immunostaining. Bar = 2 mm. (**g**) Quantitative analysis of follistatin-positive area. Five randomly selected fields of the kidney were assessed at ×100 magnification. The follistatin-positive area was measured by ImageJ 1.53a. Values are the means ± S.E. (*n* = 5–6). N.S., not significant, * *p* < 0.05 vs. 0 h. (**h**) Semiquantitative analysis of the histological changes induced by renal ischemia. The ATN area was calculated as described in Section 2. N.S., not significant, * *p* < 0.05, ** *p* < 0.01 vs. 0 h. (**i**,**j**). Correlation between urinary follistatin and follistatin-positive area (**i**) or ATN area (**j**) at 24 h after reperfusion.

Lastly, we investigated the correlation between urinary follistatin and the severity of acute kidney damage. Renal ischemia for 15, 30, and 45 min was induced in rats, and urine, kidney tissues, and serum were collected at 24 h after reperfusion. Both serum creatinine (Figure 5a) and BUN (Figure 5b) were significantly increased in rats with renal ischemia for 30 and 45 min but not 15 min. Urinary follistatin (Figure 5c) significantly increased in rats with renal ischemia for 30 and 45 min but not 15 min, which was similar to the results for urinary KIM-1 (Figure 5d). Urinary NGAL was elevated in rats with renal ischemia for 45 min but not 15 and 30 min (Figure 5e).

We also examined the expression of follistatin in kidneys with renal ischemia for the indicated periods at 24 h after reperfusion by immunostaining. In addition to the cortex, follistatin was strongly detected in the medulla of kidneys after renal ischemia for 30 and 45 min but not 15 min (Figure 5f).

Quantitative analysis demonstrated that the follistatin-positive area (Figure 5g) as well as ATN area (Figure 5h) were significantly increased in the kidney with renal ischemia for 30 and 45 min. Urinary follistatin was significantly correlated with both the follistatin-positive area (Figure 5i) and ATN area (Figure 5j), suggesting that urinary follistatin reflects the severity of acute tubular damage.

## 4. Discussion

**Figure 6:**
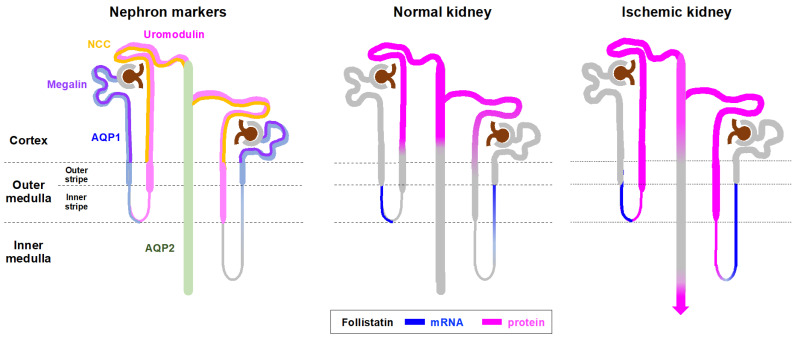
Possible mechanism of follistatin excretion into urine after renal ischemia. Aquaporin 1, AQP1; aquaporin 2, AQP2; Na-Cl co-transporter, NCC.

In the present study, we observed the difference in the distribution between follistatin mRNA and protein in the kidney. In normal kidneys, follistatin mRNA was localized in the descending limb of Henle in the outer medulla (Figure 3), and follistatin protein was mainly distributed in distal tubules of the cortex, which is downstream of its mRNA production site (Figure 1). Follistatin has a heparin-binding site and binds to heparan sulfate proteoglycan [19], a key component of the basement membrane of renal tubules [20,21]. In addition, heparinase treatment resulted in significant suppression of follistatin binding to the cell surface [22]. Taken together, it is likely that follistatin mRNA is translated into protein in the descending limb of Henle, followed by the secretion of follistatin protein into the lumen, which is sequestered by binding to heparan sulfate on the cytoplasmic surface of the distal tubules (Figure 6). We demonstrated that urinary follistatin was increased in rats with renal ischemia (Figure 4). Serum follistatin was undetectable in both normal and ischemic rats (Figure 4b), suggesting that urinary follistatin originates from free follistatin produced by renal tubules. Considering that heparan sulfate is shed and discharged into the urine under AKI [23], it is possible that free follistatin and heparan-sulfate-bound follistatin increased in the urine after renal ischemia. Further study will be required to clarify this issue.

We previously demonstrated a transient decrease in follistatin mRNA expression in the kidney after renal ischemia by Northern blot analysis [4]. In contrast, we found the upregulated expression of follistatin mRNA in the descending loop of Henle in the inner medulla after renal ischemia by in situ hybridization (Figure 3e). Real-time PCR showed that there was no significant decrease in follistatin mRNA expression after renal ischemia (Figure 3f). This discrepancy might be attributed to the difference of age of rats. Wistar rats of 200 g body weight (estimated age, 6 weeks old) was used in previous study [4]. In contrast, 8-week-old rats were used in this study. The difference of hybridization probe sequence might also affect the sensitivity of in situ hybridization, leading to the difference of signal distribution.

Previous studies demonstrated that follistatin plays a protective role under a variety of stresses [24]. The expression of follistatin is regulated transcriptionally or post-transcriptionally. The degradation rate of follistatin mRNA is also strictly controlled. Sequence analysis of the mouse follistatin promoter identified several consensus binding sites for transcription factors such as CREB, Sp1, AP-1, AP-2, Tcf, and Brachyury-T [25]. Stress triggers the binding of a transcription factor such as nuclear factor erythroid 2-related factor 2 (Nrf2) to the follistatin promoter and thereby activates follistatin transcription [26]. The WNT/β-catenin [27], MAPK-ERK-CREB [28], cAMP-dependent protein kinase (PKA), or MEK kinase (MEKK) [29] pathways are also involved in follistatin regulation. Follistatin is also important for cellular energy homeostasis and cell survival under glucose deprivation, which inhibits follistatin mRNA decay and thereby upregulates follistatin expression [30,31]. Considering that activin A stimulates follistatin mRNA expression [32], it is possible that mRNA expression of follistatin was increased by activin A in ischemic kidneys. However, this is unlikely because localization of activin A did not overlap with follistatin in ischemic kidneys (Figure 2f). In the present study, both follistatin mRNA and protein were upregulated in the inner medulla of ischemic kidneys (Figure 2 and Figure 3). A previous report showed that the thin limb of Henle in the inner medulla of the kidney became pimonidazole positive after renal ischemia [33,34], suggesting that the inner medulla is a target of hypoxic injury. If hypoxia is a potent inducer of follistatin expression in ischemic kidneys, urinary follistatin might be useful as a senser of renal hypoxia.

AKI is a common but complicated disorder associated with increased mortality and morbidity [35,36]. AKI is associated with progression to advanced chronic kidney disease. The diagnosis of AKI is usually based on increases in serum creatinine levels; however, it is less sensitive and specific for detection of acute decline of renal function in AKI. To overcome this problem, several AKI biomarkers, including NGAL, KIM-1, IL-18, L-FABP, and TIMP-2*IGFBP-7, have been identified to predict the onset of AKI, AKI severity, and renal prognosis of AKI in critically ill patients [37]. In the present study, we showed that the timing of elevation of urinary follistatin after renal ischemia was later than that of urinary NGAL or urinary L-FABP (Figure 4c). Urinary follistatin increases after renal ischemia in parallel with serum creatinine level (Figure 4a), suggesting that urinary follistatin is not suitable as an early diagnostic marker for AKI. On the other hand, we provide evidence for a significant association between urinary follistatin and AKI severity (Figure 5). Urinary follistatin levels were increased according to the duration of ischemia and was significantly correlated with follistatin-positive area as well as acute tubular damage area. These data suggest that urinary follistatin could be helpful for assessing the severity of acute kidney damage in the clinical setting. The combination of urinary follistatin with standard clinical parameters and/or established AKI biomarkers will be advantageous for monitoring of the stage of tubular recovery and assessment of the appropriate timing of intervention for patients with AKI.

## Data Availability

The datasets used and analyzed in the current study are available from the corresponding author upon reasonable request.
